# Two Splice Isoforms of *Leptinotarsa* Ecdysis Triggering Hormone Receptor Have Distinct Roles in Larva-Pupa Transition

**DOI:** 10.3389/fphys.2020.593962

**Published:** 2020-12-01

**Authors:** Chen-Hui Shen, Qing-Yu Xu, Kai-Yun Fu, Wen-Chao Guo, Lin Jin, Guo-Qing Li

**Affiliations:** ^1^ Education Ministry Key Laboratory of Integrated Management of Crop Diseases and Pests, College of Plant Protection, Nanjing Agricultural University, Nanjing, China; ^2^ Institute of Plant Protection, Xinjiang Academy of Agricultural Sciences, Urumqi, China; ^3^ Institute of Microbiological Application, Xinjiang Academy of Agricultural Science, Urumqi, China

**Keywords:** *Leptinotarsa decemlineata*, ecdysis triggering hormone receptor, isoform specificity, ecdysis behavior, larva-pupa transition

## Abstract

Insect ecdysis triggering hormone (ETH) receptors (ETHRs) are rhodopsin-like G protein-coupled receptors. Upon binding its ligand ETH, ETHR initiates a precisely programed ecdysis behavior series and physiological events. In *Drosophila melanogaster*, the *ethr* gene produces two functionally distinct splicing isoforms, *ethra* and *ethrb*. ETH/ETHRA activates eclosion hormone (EH), kinin, crustacean cardioactive peptide (CCAP), and bursicon (burs and pburs) neurons, among others, in a rigid order, to elicit the behavioral sequences and physiological actions for ecdysis at all developmental stages, whereas ETH/ETHRB is required at both pupal and adult ecdysis. However, the role of ETHRB in regulation of molting has not been clarified in any non-drosophila insects. In the present paper, we found that 20-hydroxyecdysone (20E) signaling triggers the expression of both *ethra* and *ethrb* in a Coleopteran insect pest, the Colorado potato beetle *Leptinotarsa decemlineata*. RNA interference (RNAi) was performed using double-stranded RNAs (dsRNAs) targeting the common (ds*ethr*) or isoform-specific (ds*ethra*, ds*ethrb*) regions of *ethr*. RNAi of ds*ethr*, ds*ethra*, or ds*ethrb* by the final-instar larvae arrested larva development. The arrest was not rescued by feeding 20E. All the *ethra* depleted larvae stopped development at prepupae stage; the body cavity was expanded by a large amount of liquid. Comparably, more than 80% of the *ethrb* RNAi larvae developmentally halted at the prepupae stage. The remaining *Ldethrb* hypomorphs became pupae, with blackened wings and highly-expressed *burs*, *pburs* and four melanin biosynthesis genes. Therefore, ETHRA and ETHRB play isoform-specific roles in regulation of ecdysis during larva-pupa transition in *L. decemlineata*.

## Introduction

Insects are enclosed in a sclerotized exoskeleton. For a remarkable increase in size, the integument must be shed and replaced periodically during ecdysis, a precisely timed behavior sequence that is further separated into pre-ecdysis, ecdysis, and post-ecdysis (Krüger et al., 2015; de Oliveira et al., 2019). Each of these stages correlates with major behavioral, molecular, and cellular changes (Truman, 2005). At the endocrine level, ecdysis is initiated by a near-complete discharge of ecdysis triggering hormone (ETH) from Inka cells, fueled by an endocrine positive feedback with centrally produced eclosion hormone (EH; Ewer et al., 1997; Kingan et al., 1997). ETH directly targets neurons in the central nervous system that express its receptors ETHRA and ETHRB, two functionally distinct splice variants found in many insect species (Kim et al., 2006b; Arakane et al., 2008; Dai and Adams, 2009; Roller et al., 2010; Jiang et al., 2014; Shi et al., 2017) to regulate ecdysis behavior series and ecdysis-related physiological preparations (Clark, 2004; de Oliveira et al., 2019).

In *Drosophila melanogaster*, ETHRA and ETHRB splice isoforms are known to differ both in their expression patterns within the nervous system (Kim et al., 2006a,b, 2015; Diao et al., 2016) and their affinities for the ETH peptides (Iversen et al., 2002; Park et al., 2003). The ETHRA-expressing neurons include almost all peptidergic ones, such as kinin, diuretic hormone (DH), eclosion hormone (EH), FMRFamide, crustacean cardioactive peptide (CCAP), myoinhibitory peptide (MIP), bursicon (burs/pburs heterodimer), neuropeptide F (NPF), and short neuropeptide F (sNPF; Kim et al., 2006a,b, 2015; Diao et al., 2016). In contrast, ETHRB-expressing neurons are mainly located in the dorsolateral and dorsomedial sites in each ganglion along ventral nerve cord, with a few in the brain (Kim et al., 2006b; Diao et al., 2016). Moreover, ETHRB has an around 450-fold higher affinity for ETH than ETHRA (Iversen et al., 2002; Park et al., 2003). Recently, it is discovered that the two ETHR variants functionally diverge in *D. melanogaster*. *Ethra*-expressing neurons are required for ecdysis at all developmental stages. At the time of the pupal molt, the functionally distinct *Ethra*-expressing subsets regulate not only ecdysis behavior but also fluid balance. In contrast, *ethrb*-expressing neurons are required at both pupal and adult ecdysis (Kim et al., 2015; Diao et al., 2016, 2017; Mena et al., 2016).

However, the specific functions of ETHR isoforms have only been functionally characterized by RNA interference (RNAi) in two non-drosophila insect species (Diao et al., 2016; Meiselman et al., 2017, 2018; Shi et al., 2017, 2019; Kim et al., 2018). In *Bactrocera dorsalis*, selective RNAi of *ethra*, but not *ethrb*, causes developmental failure of ecdysis at early larval stages. The dsRNA-treated larvae cannot shed the old cuticle followed by death (Shi et al., 2017). Similarly, knockdown of *ethra* rather than *ethrb* at pharate pupae in *Tribolium castaneum* results in developmental arrest at the pharate adult stage before initiation of ecdysis (Arakane et al., 2008). Given that both *ethr* transcripts are widely expressed throughout larval development stages (Meiselman et al., 2017), which role does ETHRB play during larva-pupa-adult transformation in non-drosophila insects?

In the present paper, two ETHR isoforms were identified in the Colorado potato beetle *Leptinotarsa decemlineata*. Since this beetle is sensitive to RNAi (Deng et al., 2018; Meng et al., 2018, 2019; Xu et al., 2018), we intended to clarify the isoform-specific functions of ETHR in larval development by RNAi-aided knockdown of all *ethr* variants, or each of them, and comparison of the defective phenotypes.

## Materials and Methods

### Insects

The *Leptinotarsa decemlineata* beetles were kept in an insectary according to a previously described method (Shi et al., 2013), with potato foliage at the vegetative growth or young tuber stages in order to assure sufficient nutrition. At this feeding protocol, the larvae progressed the first-, second-, penultimate-, and final-instar stages with approximate periods of 2, 2, 2, and 4 days, respectively. Upon reaching full size, the final larval instars stopped feeding and entered the wandering stage. The wandering larvae (prepupae) dropped to the ground and finally burrowed to the soil. The prepupae spent an approximately 3 days to pupate. The pupae lasted about 5 days and the adults emerged.

### Molecular Cloning

The putative *Ldethr* isoforms were obtained from the genome (Schoville et al., 2018) and transcriptome data (Shi et al., 2013) of *L. decemlineata*. The correctness of the sequences was substantiated by polymerase chain reaction (PCR) using primers in Supplementary Table S1 and Supplementary Figure S1.

### Preparation of dsRNAs

Three dsRNAs against two *Ldethr* isoforms (ds*ethr*), either *Ldethra* (ds*ethra*) or *Ldethrb* (ds*ethrb*), were designed (Supplementary Figure S1). Other dsRNAs including ds*SHD*, ds*EcR*, ds*FTZ-F1* and ds*egfp* were derived from *LdSHD*, *LdEcR*, and *LdFTZ-F1* genes from *L. decemlineata*, and enhanced green fluorescent protein (*egfp*) gene from *Aequorea victoria*. These dsRNA fragments were cloned using specific primers listed in Supplementary Table S1. All dsRNAs were individually expressed using *Escherichia coli* HT115 (DE3) competent cells lacking RNase III following the established method (Meng et al., 2019). Individual colonies were inoculated and grown until cultures reached an OD600 value of 1.0. The colonies were then induced to express dsRNA by addition of isopropyl β-D-1-thiogalactopyranoside to a final concentration of 0.1 mM. The expressed dsRNA was extracted and confirmed by electrophoresis on 1% agarose gel. Bacteria cells were centrifuged at 5000 × g for 10 min and resuspended in an equal original culture volume of 0.05 M phosphate buffered saline (PBS, pH 7.4). The bacterial solutions (at a dsRNA concentration of about 0.5 μg/ml) were used for experiment.

### Influence of Hormones on the Expression

An ecdysteroid agonist halofenozide (Hal) and 20-hydroxyecdysone (20E) were respectively purchased from ChemService (West Chester, United States) and Sigma-Aldrich (United States). Chemicals were dissolved in distilled water with added surfactant (Tween 20, 1 g/L) to give solution at the concentration of 100 ng/ml. It was further serially diluted 10-folds with distilled water if necessary.

Four independent bioassays were carried out using newly-ecdysed fourth-instar larvae. The first bioassay was to test the influence of 20E and Hal on the expression of *Ldethr* isoforms and had three treatments: (1) water (control), (2) 100 ng/ml 20E, or (3) 100 ng/ml Hal. Potato leaves were immersed for 5 s, removed, and dried for 2 h under airflow on filter paper. Five treated leaves were then placed in a Petri dish (9 cm diameter and 1.5 cm height). Ten fourth-instar larvae were confined in petri dishes containing treated leaves for 1 day as a repeat. Each treatment was replicated three times. The second to fourth bioassays were to knock down *LdSHD*, *LdEcR*, and *LdFTZ-F1* and had three treatments: (1) PBS-, (2) ds*egfp*-, (3) ds*SHD*-, ds*EcR*-, or ds*FTZ-F1*-immerged leaves. Each treatment (10 fourth-instar larvae) was replicated three times and fed for 3 days (replaced with freshly treated ones each day). The resultant larvae were used to analyze the mRNA levels of target gene and *Ldethr* variants.

### Dietary Introduction of dsRNA

The same method as previously reported was used to introduce dsRNA into larvae (Meng et al., 2019). Potato leaves were immersed with a bacterial suspension containing a dsRNA. The PBS‐ and ds*egfp*-dipped leaves were used as controls. The newly-ecdysed final-instar larvae were starved for at least 4 h prior to the experiment. Then, 10 larvae were transferred to each dish as a repeat. For each treatment, nine repeats were set and randomly allocated to three groups, each containing three replicates. The first and second groups were allowed to feed on dsRNA-dipped foliage for 3 days (replaced with freshly treated ones each day) and on untreated foliage until reaching the wandering stage. Three replicates were used to observe the defective phenotypes, pupation and adult emergence and the other three were used to extract body fluid. The third group was collected after continuously fed on treated foliage 3 days to extract total RNA.

### Rescuing Experiment by 20E

Two bioassays were carried out as previously described (Meng et al., 2019) using newly-ecdysed final-instar larvae, to test the rescuing effect of 20E at the concentration of 10 ng/ml. Ten larvae in a repeat were firstly allowed to ingest potato foliage immersed with PBS, ds*egfp* or ds*ethra* (ds*ethrb*) for 2 days, and then to consume leaves dipped with PBS, ds*egfp*, ds*ethra* (ds*ethrb*), ds*ethra* (ds*ethrb*) + 20E, for an additional day. The larvae were then transferred to untreated foliage if necessary. For each treatment, nine (for the bioassay to test rescuing effect to ds*ethra* ingestion) or six (for the bioassay to test rescuing effect to ds*ethrb* ingestion) repeats were set. Three replicates were randomly sampled for total RNA extraction 3 days after initiation of the bioassay. The other three repeats were used to observe the defective phenotypes, pupation and adult emergence. An addition of three repeats in the bioassay to test rescuing effect to ds*ethra* ingestion was used to extract body liquid (see section “Assay of Body Fluid Volume” for detail).

### Real-Time Quantitative PCR

For temporal expression analysis, RNA templates were derived from eggs, the larvae from the first through final instars, wandering larvae，pupae (5 days after burring into soil) and adults (5 days after emerging). For analysis of the tissue expression patterns, templates were from the brain-corpora cardiaca-corpora allata complex, ventral nerve cord, foregut, midgut, hindgut, Malpighian tubules, fat body, epidermis, and trachea of the day 4 fourth-instar larvae. For analysis of the effects of treatments, total RNA was extracted from treated larvae. Each sample contained at least 5–10 individuals and repeated three times. The RNA was extracted using SV Total RNA Isolation System Kit (Promega). Purified RNA was subjected to DNase I to remove any residual DNA according to the manufacturer’s instructions. Quantitative mRNA measurements were performed by real-time quantitative PCR (qRT-PCR) in technical triplicate, using four internal control genes (*LdRP4*, *LdRP18*, *LdARF1*, and *LdARF4*, the primers listed in Supplementary Table S1) according to our published results (Shi et al., 2013). An RT negative control (without reverse transcriptase) and a non-template negative control were included for each primer set to confirm the absence of genomic DNA and to check for primer-dimer or contamination in the reactions, respectively.

According to a previously described method (Bustin et al., 2009), the generation of specific PCR products was confirmed by gel electrophoresis. The primer pair for each gene was tested with a 10-fold logarithmic dilution of a cDNA mixture to generate a linear standard curve [crossing point (CP) plotted vs. log of template concentration], which was used to calculate the primer pair efficiency. All primer pairs amplified a single PCR product with the expected sizes, showed a slope less than −3.0, and exhibited efficiency values ranging from 2.3 to 2.4. Data were analyzed by the 2^−ΔΔCT^ method using the geometric mean of the four internal control genes for normalization.

### Assay of Body Fluid Volume

Twelve day after initiation of experiment, extractable body fluid volume was measured in the resultant beetles having ingested PBS, ds*egfp*, ds*ethra*, and ds*ethrb* as larvae, according to a previously described method (Diao et al., 2016), with some modifications. Briefly, the collected animals were weighed and then crushed in a pre-chilled mortar using a pellet pestle and centrifuged at 3,000 rpm in an Eppendorf micro centrifuge. The volume of fluid collected after centrifugation was measured and the ratio of this volume to the starting weight was calculated for each treatment.

### Data Analysis

We used SPSS for Windows (Chicago, IL, United States) for statistical analyses. The averages (±SE) were submitted to ANOVA with the Tukey-Kramer test.

## Results

### Sequence Alignment and Phylogenetic Analysis of *Ldethr*

**Figure 1:**
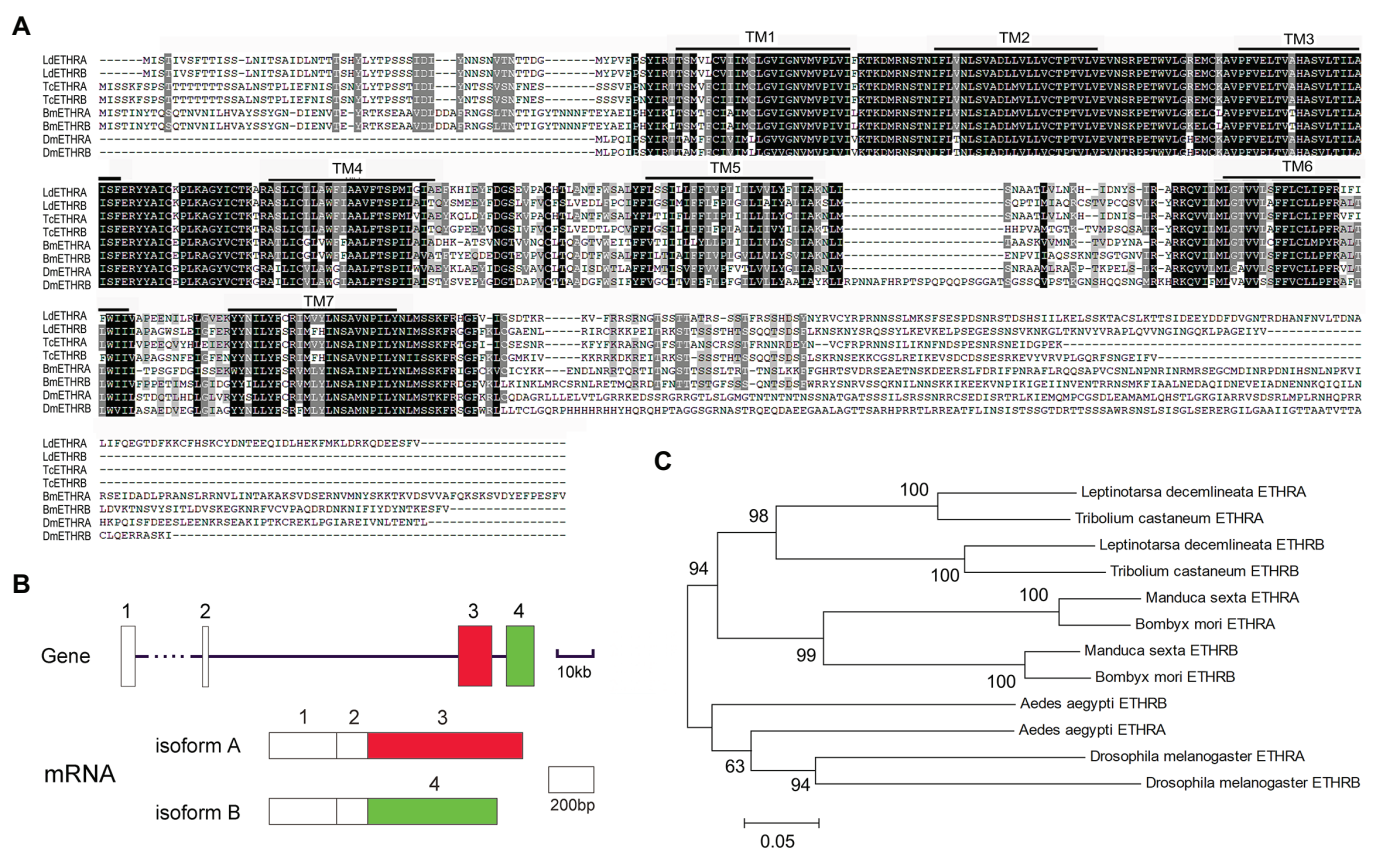
Alignment **(A)**, exon/intron structure **(B)**, and phylogenetic analysis **(C)** of ecdysis triggering hormone receptor (ETHR) derived from *Leptinotarsa decemlineata*. **(A)** Sequence alignment of *Ld*ETHR. Increasing background intensity (from light to dark) indicates an increase in sequence similarity. Gaps have been introduced to permit alignment. The transmembrane domains (TMs) are highlighted. **(B)** Boxes indicate exons. Black lines mark introns. **(C)** ETHR proteins are from two coleopteran *L. decemlineata* (ETHRA, QBH70335.1; ETHRB, QBH70336.1) and *Tribolium castaneum* (ETHRA, ABN79653; ETHRB, ABN79654), two lepidopteran *Bombyx mori* (ETHRA, AB330426; ETHRB, AB330427), *Manduca sexta* (ETHRA, AAX19163; ETHRB, AAX19164), two dipteran *Drosophila melanogaster* (ETHRA, NP_650960; ETHRB, NP_996255), and *Aedes aegypti* (ETHRA, ABI93273; ETHRB, ABI93274). The tree is constructed using the neighbor-joining method based on the full-length protein sequence alignments. Bootstrap analyses of 1,000 replications are carried out and bootstrap values >50% are shown on the tree.

By mining the genome (Schoville et al., 2018) and transcriptome data (Shi et al., 2013), two *Ldethr* transcripts were identified. Using the TMHMM server, we predicted that both proteins contained seven TMs (Figure 1A), similar to their partners from other insect species (Kim et al., 2015; de Oliveira et al., 2019). The gene structure of *Ldethr* was analyzed. *Ldethr* included four exons. The first two exons, which encode the first four of the seven TMs, were shared by both isoforms. A specific exon encoded the later three TMs specific in each *Ldethr* isoform (Figure 1B). Comparable gene structures of *ethr* genes have been documented in other insects such as *D. melanogaster* (Diao et al., 2016).

An unrooted tree was constructed to examine the phylogenetic relationships of *Ld*ETHR variants with homologous proteins from representative species across three insect orders (Coleoptera, Diptera, and Lepidoptera). In the tree, the proteins were order-specific. The two ETHR isoforms sorted with ETHR splicing variants from another coleopteran *T. castaneum*. The two ETHR variants were accordingly named as ETHRA and ETHRB (Figure 1C).

### The Expression Profiles of *Ldethr* Variants

**Figure 2:**
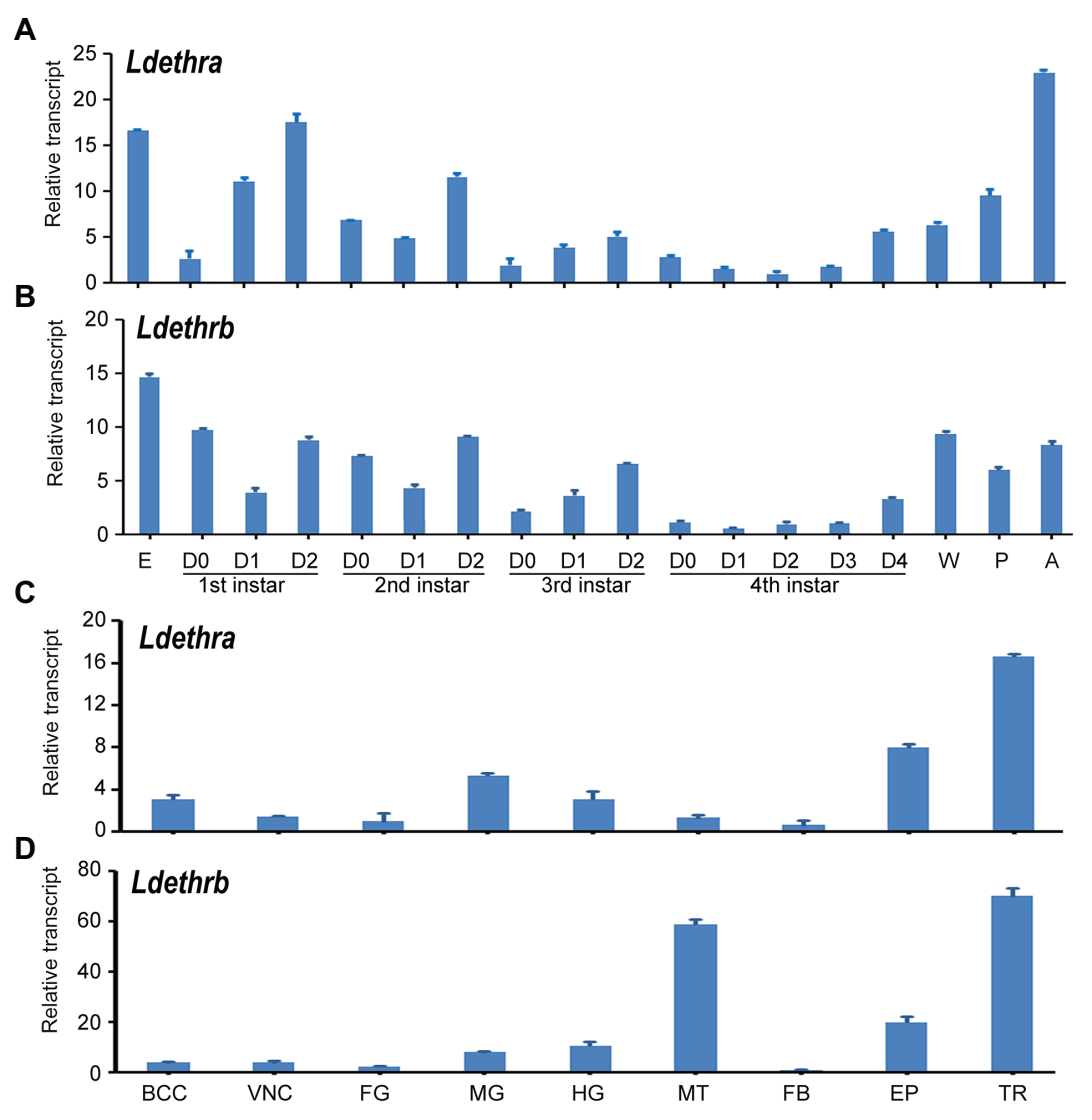
Temporal and tissue transcription patterns of *ecdysis triggering hormone receptor* (*Ldethr*) isoforms in *L. decemlineata*. For analysis of the temporal expression patterns of *Ldethra* **(A)** and *Ldethrb* **(B)**, complementary DNA templates were derived from the eggs, first, second, third, and fourth larval instars at an interval of 1 day (D0 indicated newly ecdysed larvae), wandering larvae, pupae (5 days after burring into soil) and adults (5 days after emerging). For analysis of the tissue expression patterns of *Ldethra* **(C)** and *Ldethrb* **(D)**, RNA templates were derived from the brain-corpora cardiaca-corpora allata complex (BCC), ventral nerve cord (VNC), foregut (FG), midgut (MG), hindgut (HG), Malpighian tubules (MT), fat body (FB), epidermis (EP) and trachea (TR) of the day 4 fourth-instar larvae. For each sample, three independent pools of 5–10 individuals were measured in technical triplicate using real-time quantitative PCR (qRT-PCR). The values were calculated using the 2^−ΔΔCT^ method. The lowest transcript levels are set as 1. The columns represent averages with vertical lines indicating SE.

To identify stages at which *Ldethr* isoforms are expressed and potentially required, cDNA samples prepared from the whole bodies of larvae, prepupae, pupae and sexually mature adults were analyzed by qRT-PCR. *Ldethra* and *Ldethrb* transcripts were detectable from embryo (egg) to adult by qRT-PCR. *Ldethra* was abundantly expressed at the adult and embryo; *Ldethrb* was highly transcribed at the embryo and wandering larval stages. In contrast, both *Ldethra* and *Ldethrb* were lowly expressed in the fourth-instar larval stage. Within the larval stage, copious mRNA levels of *Ldethra* and *Ldethrb* were found in the late stage of the first-, second-, third-, and fourth-instar larvae (Figures 2A,B).

Analysis of tissue expression patterns uncovered that *Ldethra* and *Ldethrb* were widely transcribed in the brain-corpora cardiaca-corpora allata complex, ventral nerve cord, foregut, midgut, hindgut, Malpighian tubules, fat body, epidermis, and trachea of the day 4 fourth-instar larvae. The levels of *Ldethra* were high in the trachea, epidermis, midgut, hindgut and brain-corpora cardiaca-corpora allata complex; while the levels of *Ldethrb* were high in the trachea, Malpighian tubules, epidermis, midgut and hindgut (Figures 2C,D).

### 20-Hydroxyecdysone Signaling Activates the Expression of *Ldethr*

**Figure 3:**
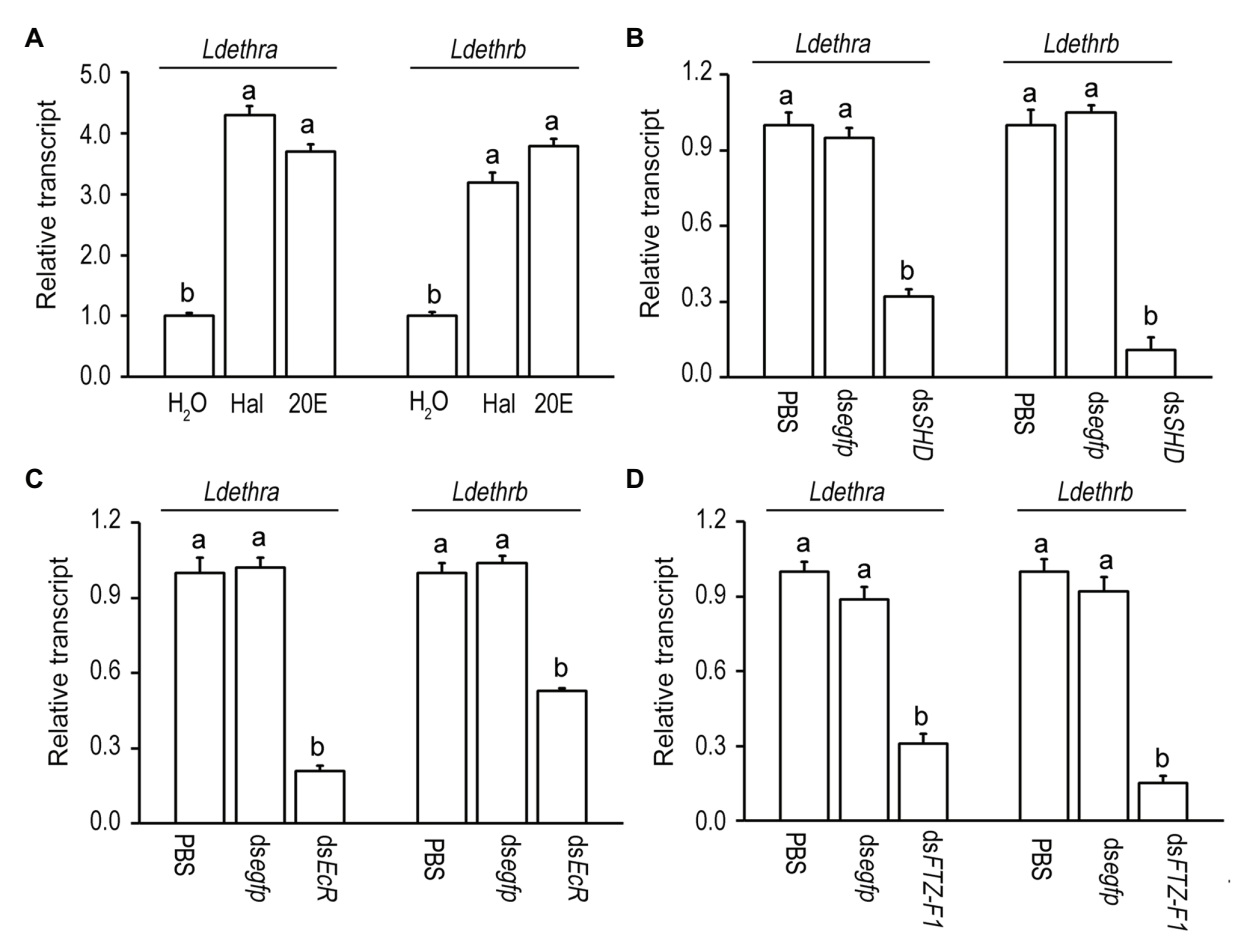
Induction of the expression of *Ldethr* isoforms by 20-hydroxyecdysone (20E) in *L. decemlineata*. For *in vivo* induction of 20E **(A)**, the newly-ecdysed fourth-instar larvae have ingested potato foliage treated with water (control), 100 ng/ml halofenozide (Hal) or 20E for 1 day. For knockdown of the genes encoding Halloween protein SHADE (SHD) **(B)**, the heterodimer of ecdysone receptor (EcR) **(C)** and a late ecdysone response gene fushi tarazu factor-1 (FTZ-F1) **(D)**, the newly-molted fourth-instar larvae had ingested ds*SHD*, ds*EcR*, and ds*FTZ-F1* dipped leaves for 3 days. The larvae having ingested PBS and ds*egfp* immersed foliage were used as controls. The values were calculated using the 2^−ΔΔCT^ method. The transcript level at control is set as 1. Different letters indicate significant difference at *p* < 0.05 compared with ANOVA and the Tukey-Kramer test.

The expression patterns uncover that the surges of mRNA levels of *Ldethra* and *Ldethrb* are correlated with the pulses of hemolymph 20E. To check whether 20E activates the transcription of *Ldethra* and *Ldethrb in vivo*, the newly-ecdysed fourth-instar larvae were allowed to feed 20E or Hal. After ingestion of 20E or Hal for 1 day, the mRNA levels of the early ecdysone-response genes *LdHR3* and *LdE75* were significantly elevated (Supplementary Figure S2). This finding indicates that *in vivo* ecdysteroid introduction activates 20E signaling. Consequently, the mRNA levels of *Ldethra* and *Ldethrb* were dramatically increased in the larvae having ingested Hal or 20E, compared with control specimens (Figure 3A).

Moreover, silencing an ecdysteroidogenesis gene Shade (*LdSHD*; Kong et al., 2014; Supplementary Figure S3A) significantly decreased the mRNA levels of *Ldethra* and *Ldethrb* (Figure 3B), compared with control specimens. In addition, depleting each of two 20E signaling genes (*LdEcR* or *LdFTZ-F1*; Liu et al., 2014; Xu et al., 2019, 2020; Supplementary Figures S3B,C) significantly reduced the mRNA levels of *Ldethra* and *Ldethrb* (Figures 3C,D).

### The *ethr* Gene Is Essential for Larva-Pupa Transition

**Figure 4:**
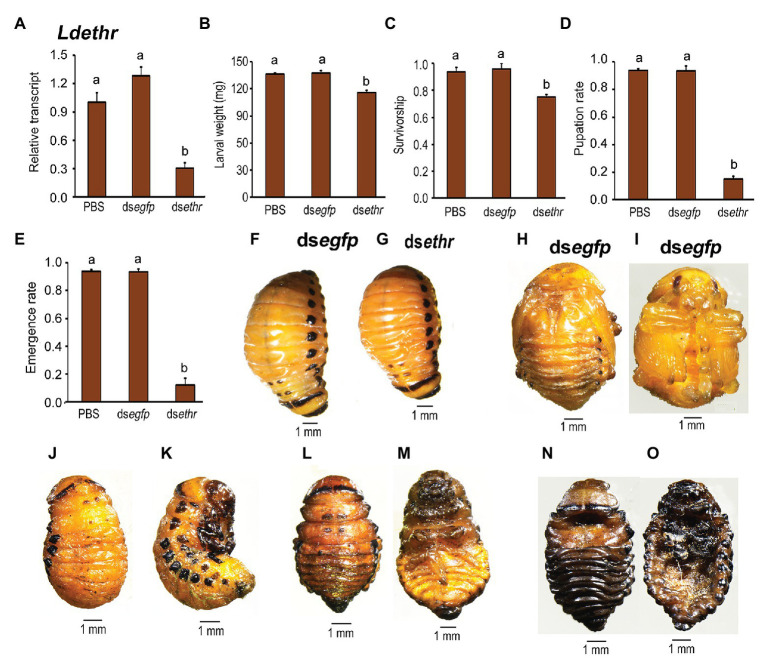
Ingestion of ds*ethr* by the final instar larvae affects larval performance in *L. decemlineata*. The newly-ecdysed fourth instar larvae had ingested PBS-, ds*egfp*-, and ds*ethr*-dipped leaves for 3 days, and untreated foliage for an addition of 1 day. Timing of the experimental procedures was shown. The expression level of *Ldethr* was measured 3 days after the initiation of the bioassay **(A)**. Relative transcripts are the ratios of relative copy numbers in treated individuals to PBS-fed controls, which is set as 1. The larvae were weighed 4 days after the initiation of the bioassay (**B,F** vs. **G**). The survivorship was observed through the fourth-instar larvae and prepupae. The pupation and emergence rates were recorded during a 4-week trial period **(C–E)**. The bars represent values (±SE). Different letters indicate significant difference at *p* < 0.05 compared with ANOVA and the Tukey-Kramer test. While the PBS‐ and ds*egfp*-fed larvae pupated 8 days **(H,I)** after initiation of bioassay, more than 80% of the *Ldethr* RNAi larvae remain as prepupae **(D)** 10 **(J,K)** and 15 **(L,M)** days after initiation of bioassay, completely wrapped in the old larval exuviae. These prepupae become withered, dried and darkened gradually, and finally die in soil **(N,O)**.

To characterize ETHR function, we took advantage of RNAi by immersing the potato foliage with ds*ethr* targeting the common sequence of both *Ldethr* variants. The results were examined according to the experimental procedure timing listed in Supplementary Figure S4. Ingesting ds*ethr* significantly declined the target mRNA level (Figure 4A). Larval growth was inhibited in the ds*ethr* fed beetles (Figures 4B,F vs. G). Defective phenotypes were noted during larva-pupa-adult transition. The most severe defect occurred in around 22% of the *Ldethr* RNAi larvae. These animals remained on the soil surface, gradually dried and finally died within 10 days (Figure 4C).

The remaining 78% of the ds*ethr*-treated larvae showed moderate or slight defective phenotypes. They displayed similar wandering behavior and dug into soil normally after developing a similar period to those in the ds*egfp*‐ and PBS-fed larvae. More than 80% of these *Ldethr* RNAi prepupae mined into soil exhibited a modest defect. They failed to pupate (Figure 4D) 10 (Figures 4J,K) and 15 (Figures 4L,M) days after initiation of bioassay, wrapped completely in the old larval exuviae, unlike the ds*egfp*‐ and PBS-fed larvae pupating 8 days after initiation of bioassay (Figures 4D,H,I). These prepupae gradually darkened and withered, and finally died (Figures 4N,O).

Lastly, less than 20% of these *Ldethr* RNAi prepupae that mined into soil displayed a minor defect. They normally pupated and emerged as adults (Figures 4D,E). However, all the *Ldethr* RNAi adults eventually died in a week after emergence.

### Two Splice ETHR Variants Have Distinct Roles in Larva-Pupa Transition

**Figure 5:**
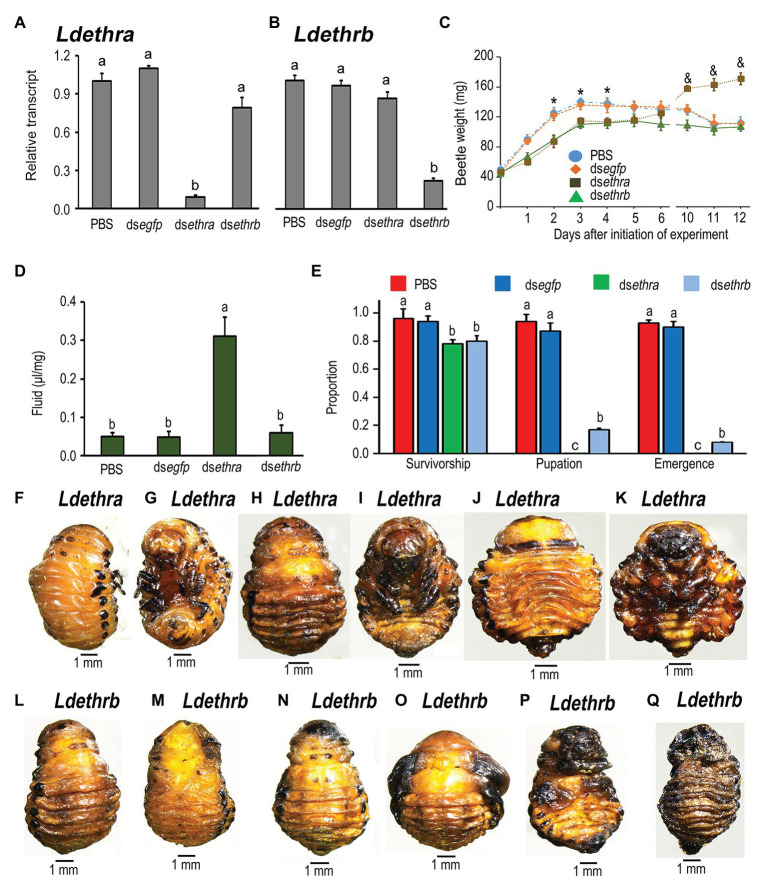
Two splice ETHR isoforms have distinct roles in larva-pupa transition in *L. decemlineata*. The newly-ecdysed fourth instar larvae had ingested PBS-, ds*egfp*-, ds*ethra-* and ds*ethrb*-immersed potato foliage for 3 days, and untreated foliage for an addition of 1 day. The expression levels of *Ldethra* and *Ldethrb* were tested 3 days after the initiation of the bioassay **(A,B)**. Relative transcripts are the ratios of relative copy numbers in treated individuals to PBS-fed controls, which are set as 1. The larvae were weighed 1–6 and 10–12 days after the initiation of the bioassay **(C)**. The extracellular fluid volumes were determined 12 days after the initiation of the bioassay **(D)**. The survivorship, pupation and emergence rates were recorded during a 4-week trial period **(E)**. The bars represent values (±SE). Different letters indicate significant difference at *p* < 0.05 compared with ANOVA and the Tukey-Kramer test. The defective phenotypes in prepupae or pupae having ingested ds*ethra* **(F–K)** or ds*ethrb* **(L–Q)** as larvae are shown.

In the larvae having fed for 3 days on foliage immersed with an isoform-specific dsRNA (ds*ethra* or ds*ethrb*), the mRNA level of target transcript was significantly decreased. In contrast, ingestion either ds*ethra* or ds*ethrb* did not affect the transcript level of non-target isoform (Figures 5A,B).

Ingestion of ds*ethra* or ds*ethrb* suppressed larval growth (Figure 5C). The fresh weights of the resulting larvae having fed on either ds*ethra* or ds*ethrb* were significantly lighter than those of PBS‐ and ds*egfp*-fed larvae 2, 3, and 4 days after initiation of bioassay. When the larvae entered prepupae stages (5 and 6 days after experiment), the difference in body weights became smaller (Figure 5C). Similar to knockdown of two *ethr* isoforms, consumption of either ds*ethra* or ds*ethrb* caused various defective phenotypes, varying in degree. Firstly, around 20% larval mortalities were noted before entering the soil (Figure 5E). Secondly, although the remaining approximately 80% ds*ethra*‐ or ds*ethrb*-treated larvae displayed similar wandering behavior and dug into soil normally after a similar developing period to those in the ds*egfp*‐ and PBS-fed larva, feeding ds*ethra* or ds*ethrb* arrested development.

In contrast to the ds*egfp*‐ and PBS-fed larvae that pupated 8 days after initiation of bioassay (Figure 5E), all *Ldethra* depleted larvae remained as prepupae (Figures 5E–I), completely wrapped in the old larval exuviae. The fresh weights of the resultant prepupae were greatly increased 10, 11, and 12 days after initiation of bioassay (Figure 5C). At the same time, the body cavity was expanded. A large amount of liquid was found in the cavity (Figures 5J,K). We assayed the accumulation of extracellular fluid by directly measuring the extractable volume of liquid normalized to body weight [liquid volume (μl) per mg fresh weight]. We found that fluid accumulation was significantly elevated 12 days after the initiation of bioassay (Figure 5D).

As for RNAi of *Ldethrb*, approximately 80% of the resulting larvae were developmentally halted at the prepupae stage (Figures 5L–N), completely or partially wrapped in the old larval cuticles. These prepupae gradually darkened and dried, and finally died (Figures 5P,Q). Around 20% of the *Ldethrb* hypomorphs shed their larval exuvia and became pupae, with blackened wings (Figures 5E,O), in contrast to control pupae (Figures 4H,I).

**Figure 6:**
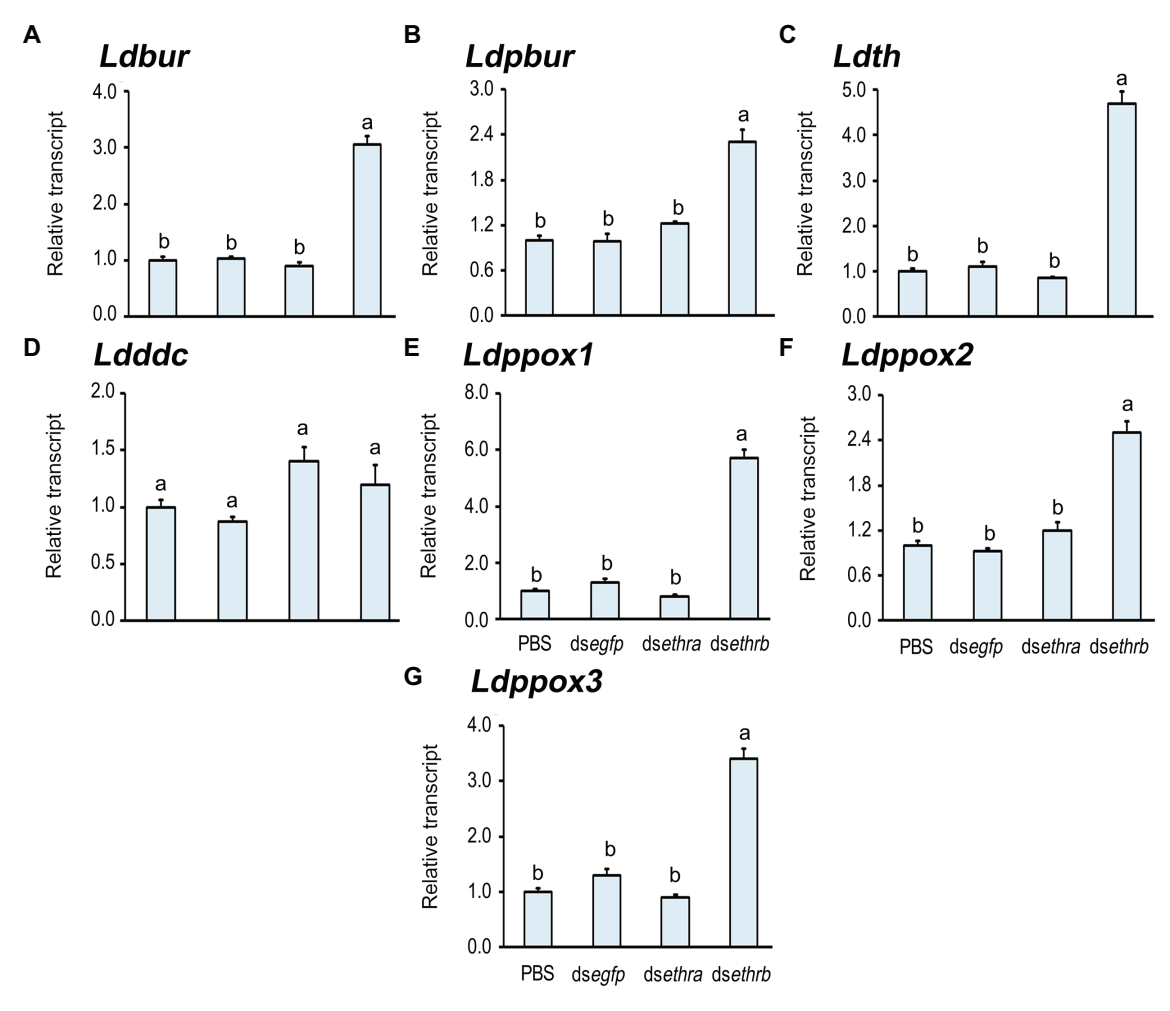
Knockdown of *ethrb* isoform prematurely activates melanin biosynthesis in *L. decemlineata*. The newly-ecdysed fourth instar larvae had ingested PBS-, ds*egfp*-, ds*ethra-*, and ds*ethrb*-immersed potato foliage for 3 days, and untreated foliage for an addition of 1 day. The expression levels of *Ldbur*, *Ldpbur*, *Ldth*, *Ldddc*, *Ldppox1*, *Ldppox2*, and *Ldppox3* were tested 12 days after the initiation of the bioassay **(A–G)**. Relative transcripts are the ratios of relative copy numbers in treated individuals to PBS-fed controls, which are set as 1. Different letters indicate significant difference at *p* < 0.05 compared with ANOVA and the Tukey-Kramer test.

The darkened wings indicate the deposition of dark black and brown melanins in wings, a process induced by bursicon heterodimer (burs/pburs; Kim et al., 2006a,b, 2015; Diao et al., 2016). The biosynthesis of melanin pigments from tyrosine is catalyzed by tyrosine hydroxylase (TH), dopa decarboxylase (DDC), and prophenol oxidase (PPO), among others (Radovic et al., 2002; Protas and Patel, 2008; Wittkopp et al., 2009; Wittkopp and Beldade, 2009; Kronforst et al., 2012; Takahashi, 2013; Massey and Wittkopp, 2016). We accordingly tested the expression level of *Ldbur*, *Ldpbur*, *Ldth*, *Ldddc*, *Ldppox1*, *Ldppox2*, and *Ldppox3* in these wing-darkened pupae. As expected, the expression levels of *Ldbur*, *Ldpbur*, *Ldth*, *Ldppox1*, *Ldppox2*, and *Ldppox3* were increased compared with those in the control pupae (Figure 6).

A few *Ldethrb* RNAi pupae with darkened wings finally emerged as adults (Figure 5E). All these *Ldethrb* RNAi adults appeared normal in shape, but eventually died within 1 week after eclosion.

### Rescuing Effect by 20E in the *Ldethra* or *Ldethrb* RNAi Final Instar Larvae

**Figure 7:**
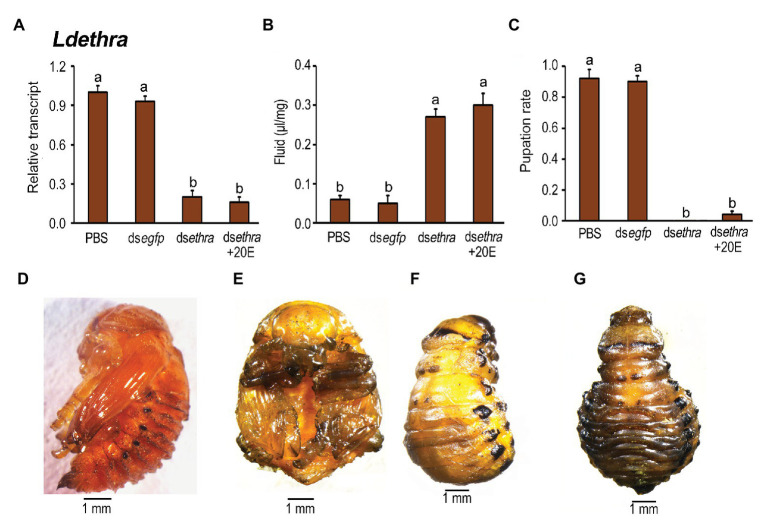
The rescuing effects of 20E on pupation in the *Ldethra* RNAi larvae in *L. decemlineata*. The newly-ecdysed final instar larvae had ingested PBS, ds*egfp*, ds*ethra*-, and ds*ethra* + 20E-dipped leaves. The expression level of *Ldethra* was tested **(A)**. Relative transcript levels are the ratios of relative copy numbers in treated individuals to PBS-fed controls, which is set as 1. The extracellular fluid volumes were determined 12 days after the initiation of the bioassay **(B)**. The pupation rate was recorded during a 4-week trial period **(C)**. The bars represent values (±SE). Different letters indicate significant difference at *p* < 0.05 compared with ANOVA and the Tukey-Kramer test. The prepupa defective phenotypes that have fed on ds*ethra* + 20E as larvae are shown **(F,G)**, compared with that in control **(D,E)**.

20E ingestion by *Ldethra* RNAi larvae did not rescue the decreased expression levels of *Ldethra* (Figure 7A). Moreover, 20E introduction neither lessened the accumulated extracellular fluid (Figure 7B) nor elevated the low pupation rate (Figure 7C). While the ds*egfp*‐ and PBS-fed larvae pupated 8 days after initiation of bioassay (Figures 7D,E), almost all of the resultant larvae ingested ds*ethra* and ds*ethra* + 20E developmentally arrested at prepupae stage (Figure 7F). Furthermore, 10, 11, and 12 days after initiation of bioassay, the body cavity of the *Ldethra* hypomorph was expanded, especially at the abdomen (Figure 7G).

**Figure 8:**
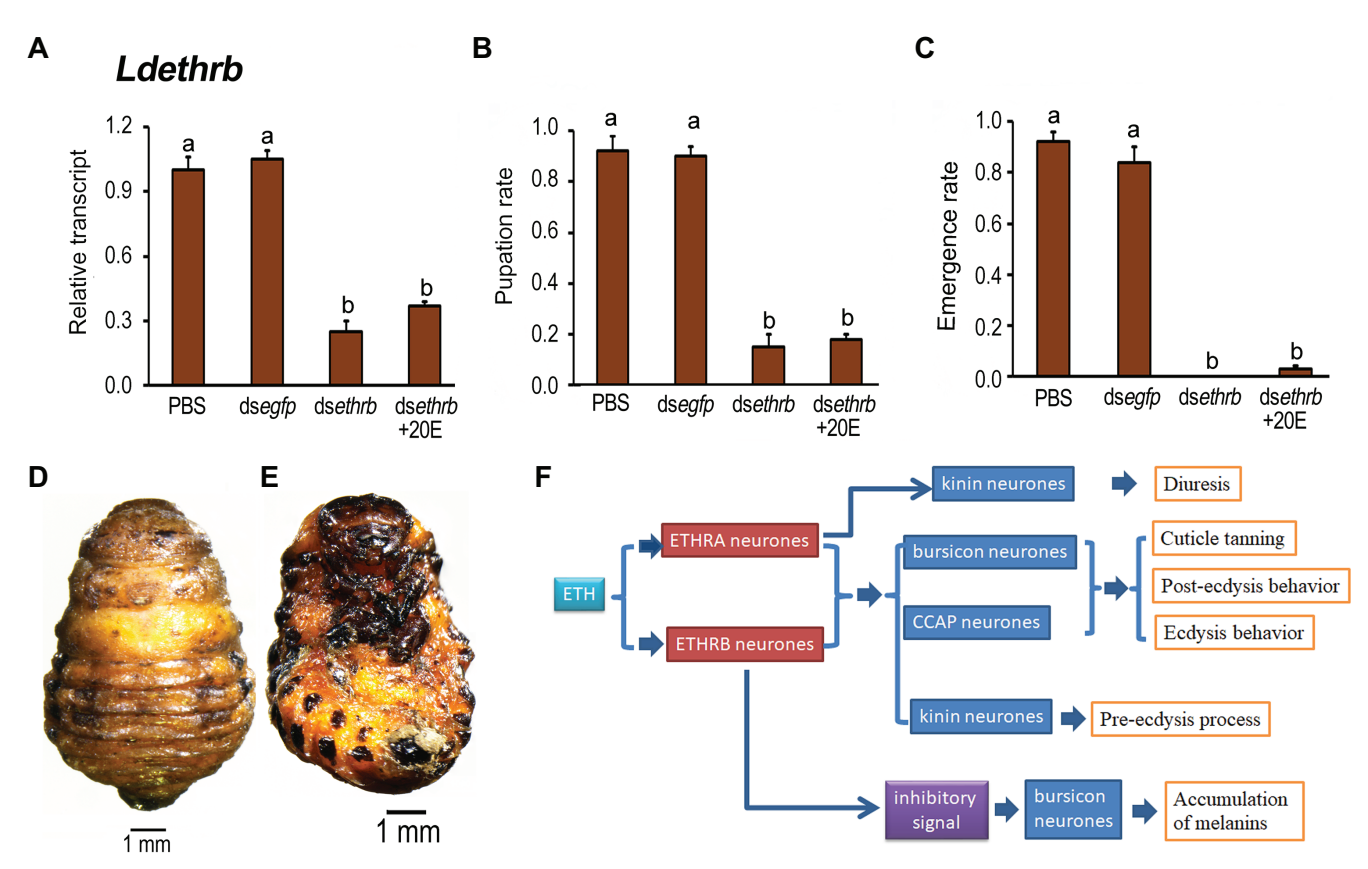
The rescuing effects of 20-hydroxyecdysone (20E) on pupation in the *Ldethrb* RNAi larvae in *L. decemlineata*. The newly-ecdysed final instar larvae had ingested PBS, ds*egfp*, ds*ethrb*-, and ds*ethrb* + 20E-immersed potato foliage. The expression level of *Ldethrb* was determined **(A)**. Relative transcript levels are the ratios of relative copy numbers in treated individuals to PBS-fed controls, which is set as 1. The pupation and emergence rates were observed during a 4-week trial period **(B,C)**. The bars represent values (±SE). Different letters indicate significant difference at *p* < 0.05 compared with ANOVA and the Tukey-Kramer test. The prepupa defective phenotypes that have fed on ds*ethrb* + 20E as larvae are given **(D,E)**. A model depicts the combinatory role of ETHRA and ETHRB on regulation of ecdysis in *L. decemlineata* (see section “Discussion” for details) **(F)**.

We repeated the rescuing experiment in *Ldethrb* RNAi larvae using 20E and found that ingestion of 20E did not restore the lowed expression level of *Ldethrb* (Figure 8A), nor alleviated the impairment of pupation and adult eclosion (Figures 8B,C). Around 80% of the resultant larvae ingested ds*ethrb* and ds*ethrb* + 20E remained developmentally stationary in the prepupal stage (Figures 8D,E).

The rescuing findings reveal that both ETHRA and ETHRB act downstream of 20E biosynthesis.

## Discussion

Ecdysis process consists of behavioral routines and physiological events that are expressed in a specific sequence. In *D. melanogaster*, the sequential nature of ecdysis is based on the progressive activation of different ETH targets: the ETHRB‐ and ETHRA-expressing neurons (Kim et al., 2015; Diao et al., 2016, 2017; Mena et al., 2016). In this survey, we found that alternative use of exon causes different ETHR variants, ETHRA and ETHRB, in *L. decemlineata* (Figure 1). RNAi-aided knockdown of both *Ldethr* transcripts in *L. decemlineata* (Figure 4) phenocopied ETH depletion insects such as *D. melanogaster* (Park et al., 2002; Cho et al., 2014), *B. dorsalis* (Shi et al., 2017), and *Schistocerca gregaria* (Lenaerts et al., 2017), indicating that the *ethr* gene encodes the sole receptor for ETH in mediating ecdysis in *L. decemlineata*. In this survey, we focused on the isoform-specific roles of the two splicing variants in larva-pupa transition in *L. decemlineata*.

### Both *Ldethr* Isoforms Are Activated by 20E Signal

In this study, we found that the expression peaks of either *Ldethra* or *Ldethrb* in first through fourth larval instars exhibited a clear correlation with 20E pulses just before ecdysis (Figure 2). Consistent with our result, the peaks in *ethr* transcript levels are observed together with the peaks in ecdysteroid titers in *M. sexta*, *A. aegypti*, and *S. gregaria* (Kim et al., 2006b; Dai and Adams, 2009; Lenaerts et al., 2017). Based on the above researches in both holometabolans and hemimetabolans, the temporal expression profiles of both *Ldethra* and *Ldethrb* suggest that they may regulate by an ecdysteroid pulse.

Consistent with the suggestion, we found that ingestion of either Hal or 20E augmented the expression of both *Ldethra* and *Ldethrb*, whereas knockdown of *SHD* decreased their transcription in *L. decemlineata*. Furthermore, transcript levels of both *Ldethra* and *Ldethrb* were significantly reduced in *LdEcR* or *LdFTZ-F1* knockdown fourth larval instars, compared to control beetles (Figure 3). In agreement with our results, 20E regulates *ethr* expression in the larval stages of moths, mosquitoes, desert locust and *D. melanogaster* adults (Žitňan et al., 1999, 2007; Dai and Adams, 2009; Lenaerts et al., 2017; Meiselman et al., 2017).

The findings that the transcript levels of both *Ldethra* and *Ldethrb* are highly dependent on functional 20E signaling indicate that both variants are essential for larva metamorphoses in *L. decemlineata*. Conversely, in *B. dorsalis*, only *ethra* displays a clear correlation with 20E titer. As expected, only ETHRA is responsible for the regulation of larva-pupa metamorphosis; whereas ETHRB control adult ovary growth (Shi et al., 2017, 2019).

### ETHRA Is Essential for Larva-Pupa Ecdysis

In this survey, we uncovered that feeding *ethra* at the final instar stage impaired larva-pupa ecdysis. The defects could not be rescued by 20E. All the *ethra* hypomorphs were developmentally halted at the prepupae stage in *L. decemlineata* (Figures 5, 7). Consistent with our results, suppression of ETHRA-expressing neurons leads to 100% larval lethality in *D. melanogaster*, with ecdysis deficits; restricting suppression of ETHRA-expressing neurons at the pupal and pharate adult stages blocks adult ecdysis and results in 100% lethality (Diao et al., 2016). Similar results have been documented using RNAi in holometabolans including *B. dorsalis* (Shi et al., 2017) and *T. castaneum* (Arakane et al., 2008), hemimetabolans such as *S. gregaria* (Lenaerts et al., 2017) and other arthropods for instance *Macrobrachium nipponense* (Liang et al., 2017).

An obvious defective phenotype was the accumulation of extracellular liquid in the *ethra* depletion prepupae in *L. decemlineata*, which resulted in the expanded bodies 10, 11, and 12 days after initiation of bioassay (Figure 5). It is well known that ETHRA-expressing neurons include kinin-, EH-, CCAP-, and bursicon-producing ones that regulate different phases of the ecdysis sequence (Kim et al., 2006a,b, 2015; Diao et al., 2016). In *D. melanogaster*, kinin regulates fluid balance by targeting Malpighian tubules (Terhzaz et al., 1999). Suppression of ETHRA within all kinin-expressing neurons accumulates a larger amount of water in the resultant pupae (Diao et al., 2016).

The similar defective phenotypes demonstrate that the critical role of ETHRA during larva-pupa transition is conserved among dipterans such as *D. melanogaster* (Kim et al., 2006a,b, 2015; Diao et al., 2016) and *B. dorsalis* (Shi et al., 2017), and coleopterans such as *T. castaneum* (Arakane et al., 2008) and *L. decemlineata* (this study).

### ETHRB Is Required for Larva-Pupa Transition

In the present paper, our results revealed that ETHRB acts downstream of 20E biosynthesis to regulate larva-pupa molting in *L. decemlineata* (Figures 5, 8). Similar role of ETHRB has been reported in *D. melanogaster* (Diao et al., 2016), but not in *B. dorsalis* (Shi et al., 2017) and *T. castaneum* (Arakane et al., 2008).

In this study, we established that some *Ldethrb* RNAi pupae had blackened wings (Figure 5), a phenotype caused by the accumulation of melanins (Radovic et al., 2002; Protas and Patel, 2008; Wittkopp et al., 2009; Wittkopp and Beldade, 2009; Kronforst et al., 2012; Takahashi, 2013; Massey and Wittkopp, 2016). Moreover, we uncovered that the expression levels of *Ldbur*, *Ldpbur*, *Ldth*, *Ldppox1*, *Ldppox2*, and *Ldppox3* were upraised (Figure 6). Our results provide, for the first time, a compelling piece of experimental evidence that the inhibitory inputs from ETHRB-expressing neurons indirectly act on bursicon neurons to regulate the production and secretion of burs and pburs. During wandering stage, *Ldethrb* was highly expressed. The resultant ETHRB acts as an indirect inhibitor of bursicon-producing neurons. Bursicon does not release until after adult emergence. In the present paper, depletion of ETHRB by RNAi lessens these inhibitory inputs, burs and pburs that are prematurely expressed and released, the melanins are precociously biosynthesized and accumulated in the pupal wings in *L. decemlineata*. This issue deserves further research to clarify.

In summary, our results establish the isoform-specific roles of ETHRA and ETHRB on regulation of ecdysis in *L. decemlineata*. Accordingly, we propose a model summarizing these findings (Figure 8F). During larva-pupa transition, ETH regulates ecdysis behavior series and cuticle tanning by triggering ETHRA and ETHRB to coordinately activate kinin, CCAP, and bursicon neurons. Moreover, the ETH/ETHRA way specifically controls kinin signal for fluid balance by targeting Malpighian tubules; whereas ETH/ETHRB inhibitory signal is critical for suppression of the premature expression and release of burs and pburs in bursicon neurons.

## Data Availability Statement

The original contributions presented in the study are included in the article/Supplementary Material, further inquiries can be directed to the corresponding author.

## Author Contributions

G-QL, LJ, C-HS, and Q-YX conceived the study. LJ, C-HS, Q-YX, and G-QL participated in the design of the experiments and the interpretation of the results. C-HS, Q-YX, K-YF, W-CG, and LJ performed the experiments. G-QL, C-HS, and LJ wrote the first draft of the manuscript. All authors contributed to the article and approved the submitted version.

### Conflict of Interest

The authors declare that the research was conducted in the absence of any commercial or financial relationships that could be construed as a potential conflict of interest.

## References

[ref1] Arakane Y. Li B. Muthukrishnan S. Beeman R. W. Kramer K. J. Park Y. (2008). Functional analysis of four neuropeptides, EH, ETH, CCAP and bursicon, and their receptors in adult ecdysis behavior of the red flour beetle, *Tribolium castaneum*. Mech. Dev. 125, 984–995. 10.1016/j.mod.2008.09.002, PMID: 18835439

[ref2] Bustin S. A. Benes V. Garson J. A. Hellemans J. Huggett J. Kubista M.. (2009). The MIQE guidelines: minimum information for publication of quantitative real-time PCR experiments. Clin. Chem. 55, 611–622. 10.1373/clinchem.2008.112797, PMID: 19246619

[ref3] Cho K. H. Daubnerová I. Park Y. Žitnan D. Adams M. E. (2014). Secretory competence in a gateway endocrine cell conferred by the nuclear receptor βFTZ-F1 enables stage-specific ecdysone responses throughout development in *Drosophila*. Dev. Biol. 385, 253–262. 10.1016/j.ydbio.2013.11.003, PMID: 24247008 PMC3900412

[ref4] Clark A. C. (2004). Neuroendocrine control of larval ecdysis behavior in *Drosophila*: complex regulation by partially redundant neuropeptides. J. Neurosci. 24, 4283–4292. 10.1523/JNEUROSCI.4938-03.2004, PMID: 15115824 PMC6729283

[ref5] Dai L. Adams M. E. (2009). Ecdysis triggering hormone signaling in the yellow fever mosquito *Aedes aegypti*. Gen. Comp. Endocrinol. 162, 43–51. 10.1016/j.ygcen.2009.03.004, PMID: 19298818 PMC2851739

[ref6] de Oliveira A. L. Calcino A. Wanninger A. (2019). Ancient origins of arthropod moulting pathway components. eLife 8:e46113. 10.7554/eLife.46113, PMID: 31266593 PMC6660194

[ref7] Deng P. Xu Q. -Y. Fu K. -Y. Guo W. -C. Li G. -Q. (2018). RNA interference against the putative insulin receptor substrate gene chico affects metamorphosis in *Leptinotarsa decemlineata*. Insect Biochem. Mol. Biol. 103, 1–11. 10.1016/j.ibmb.2018.10.001, PMID: 30296480

[ref8] Diao F. Elliott A. D. Diao F. Shah S. White B. H. (2017). Neuromodulatory connectivity defines the structure of a behavioral neural network. eLife 6:e29797. 10.7554/eLife.29797, PMID: 29165248 PMC5720592

[ref9] Diao F. Mena W. Shi J. Park D. Diao F. Taghert P.. (2016). The splice isoforms of the *Drosophila* ecdysis triggering hormone receptor have developmentally distinct roles. Genetics 202, 175–189. 10.1534/genetics.115.182121, PMID: 26534952 PMC4701084

[ref10] Ewer J. Gammie S. C. Truman J. W. (1997). Control of insect ecdysis by a positive-feedback endocrine system: roles of eclosion hormone and ecdysis triggering hormone. J. Exp. Biol. 200, 869–881. PMID: 9100362 10.1242/jeb.200.5.869

[ref11] Iversen A. Cazzamali G. Williamson M. Hauser F. Grimmelikhuijzen C. J. P. (2002). Molecular identification of the first insect ecdysis triggering hormone receptors. Biol. Biophys. Res. Commun. 299, 924–931. 10.1016/S0006-291X(02)02798-5 12470668

[ref12] Jiang H. Wei Z. Nachman R. J. Adams M. E. Park Y. (2014). Functional phylogenetics reveals contributions of pleiotropic peptide action to ligand-receptor coevolution. Sci. Rep. 4:6800. 10.1038/srep06800, PMID: 25348027 PMC4210869

[ref13] Kim D. H. Han M. R. Lee G. Lee S. S. Kim Y. J. Adams M. E. (2015). Rescheduling behavioral subunits of a fixed action pattern by genetic manipulation of peptidergic signaling. PLoS Genet. 11:e1005513. 10.1371/journal.pgen.1005513, PMID: 26401953 PMC4581697

[ref14] Kim D. H. Kim Y. J. Adams M. E. (2018). Endocrine regulation of airway clearance in *Drosophila*. Proc. Natl. Acad. Sci. U. S. A. 115, 1535–1540. 10.1073/pnas.1717257115, PMID: 29386394 PMC5816185

[ref15] Kim Y. J. Zitnan D. Cho K. H. Schooley D. A. Mizoguchi A. Adams M. E. (2006b). Central peptidergic ensembles associated with organization of an innate behavior. Proc. Natl. Acad. Sci. U. S. A. 103, 14211–14216. 10.1073/pnas.0603459103, PMID: 16968777 PMC1599936

[ref16] Kim Y. J. Zitnan D. Galizia C. G. Cho K. H. Adams M. E. (2006a). A command chemical triggers an innate behavior by sequential activation of multiple peptidergic ensembles. Curr. Biol. 16, 1395–1407. 10.1016/j.cub.2006.06.027, PMID: 16860738

[ref17] Kingan T. G. Gray W. Žitnan D. Adams M. E. (1997). Regulation of ecdysis-triggering hormone release by eclosion hormone. J. Exp. Biol. 200, 3245–3256. PMID: 9364030 10.1242/jeb.200.24.3245

[ref18] Kong Y. Liu X. -P. Wan P. -J. Shi X. -Q. Guo W. -C. Li G. -Q. (2014). The P450 enzyme shade mediates the hydroxylation of ecdysone to 20-hydroxyecdysone in the colorado potato beetle, *Leptinotarsa decemlineata*. Insect Mol. Biol. 23, 632–643. 10.1111/imb.12115, PMID: 24989229

[ref19] Kronforst M. R. Barsh G. S. Kopp A. Mallet J. Monteiro A. Mullen S. P.. (2012). Unraveling the thread of nature’s tapestry: the genetics of diversity and convergence in animal pigmentation. Pigment Cell Melanoma Res. 25, 411–433. 10.1111/j.1755-148X.2012.01014.x, PMID: 22578174

[ref20] Krüger E. Mena W. Lahr E. C. Johnson E. C. Ewer J. (2015). Genetic analysis of eclosion hormone action during *Drosophila* larval ecdysis. Development 142, 4279–4287. 10.1242/dev.126995, PMID: 26395475

[ref21] Lenaerts C. Cools D. Verdonck R. Verbakel L. Vanden Broeck J. Marchal E. (2017). The ecdysis triggering hormone system is essential for successful moulting of a major hemimetabolous pest insect, *Schistocerca gregaria*. Sci. Rep. 7:46502. 10.1038/srep46502, PMID: 28417966 PMC5394484

[ref22] Liang G. X. Fu H. T. Qiao H. Sun S. M. Zhang W. Y. Jin S. B. (2017). Molecular cloning and characterization of a putative ecdysis-triggering hormone receptor (ETHR) gene from *Macrobrachium nipponense*. J. World Aquacult. Soc. 49, 1081–1094. 10.1111/jwas.12451

[ref23] Liu X. -P. Fu K. -Y. Lü F. -G. Meng Q. -W. Guo W. -C. Li G. -Q. (2014). Involvement of FTZ-F1 in the regulation of pupation in *Leptinotarsa decemlineata* (say). Insect Biochem. Mol. Biol. 55, 51–60. 10.1016/j.ibmb.2014.10.008, PMID: 25446391

[ref24] Massey J. H. Wittkopp P. J. (2016). The genetic basis of pigmentation differences within and between *Drosophila* species. Curr. Top. Dev. Biol. 119, 27–61. 10.1016/bs.ctdb.2016.03.004, PMID: 27282023 PMC5002358

[ref25] Meiselman M. R. Kingan T. G. Adams M. E. (2018). Stress-induced reproductive arrest in *Drosophila* occurs through ETH deficiency-mediated suppression of oogenesis and ovulation. BMC Biol. 16:18. 10.1186/s12915-018-0484-9, PMID: 29382341 PMC5791332

[ref26] Meiselman M. Lee S. S. Tran R. T. Dai H. Ding Y. Rivera-Perez C.. (2017). Endocrine network essential for reproductive success in *Drosophila melanogaster*. Proc. Natl. Acad. Sci. U. S. A. 114, E3849–E3858. 10.1073/pnas.1620760114, PMID: 28439025 PMC5441734

[ref27] Mena W. Diegelmann S. Wegener C. Ewer J. (2016). Stereotyped responses of *Drosophila* peptidergic neuronal ensemble depend on downstream neuromodulators. eLife 5:e19686. 10.7554/eLife.19686, PMID: 27976997 PMC5158135

[ref28] Meng Q. -W. Xu Q. -Y. Deng P. Fu K. -Y. Guo W. -C. Li G. -Q. (2018). Involvement of methoprene-tolerant (Met) in the determination of the final body size in *Leptinotarsa decemlineata* (Say) larvae. Insect Biochem. Mol. Biol. 97, 1–9. 10.1016/j.ibmb.2018.04.003, PMID: 29680288

[ref29] Meng Q. -W. Xu Q. -Y. Zhu T. -T. Jin L. Fu K. -Y. Guo W. -C.. (2019). Hormonal signaling cascades required for phototaxis switch in wandering *Leptinotarsa decemlineata* larvae. PLoS Genet. 15:e1007423. 10.1371/journal.pgen.1007423, PMID: 30615614 PMC6336328

[ref30] Park Y. Kim Y. J. Dupriez V. Adams M. E. (2003). Two subtypes of ecdysistriggering hormone receptor in *Drosophila melanogaster*. J. Biol. Chem. 278, 17710–17715. 10.1074/jbc.M301119200, PMID: 12586820

[ref31] Park Y. Valery F. Gill S. S. Adams M. E. (2002). Deletion of the ecdysis triggering hormone gene leads to lethal ecdysis deficiency. Development 129, 493–503. PMID: 11807040 10.1242/dev.129.2.493

[ref32] Protas M. E. Patel N. H. (2008). Evolution of coloration patterns. Annu. Rev. Cell Dev. Biol. 24, 425–446. 10.1146/annurev.cellbio.24.110707.175302, PMID: 18593352

[ref33] Radovic A. Wittkopp P. J. Long A. D. Drapeau M. D. (2002). Immunohistochemical colocalization of yellow and male-specific fruitless in *Drosophila melanogaster* neuroblasts. Biochem. Biophys. Res. Commun. 293, 1262–1264. 10.1016/S0006-291X(02)00366-2, PMID: 12054512

[ref34] Roller L. Zitnanová I. Dai L. Simo L. Park Y. Satake H.. (2010). Ecdysis triggering hormone signaling in arthropods. Peptides 31, 429–441. 10.1016/j.peptides.2009.11.022, PMID: 19951734 PMC2854297

[ref35] Schoville S. D. Chen Y. H. Andersson M. N. Benoit J. B. Bhandari A. Bowsher J. H.. (2018). A model species for agricultural pest genomics: the genome of the colorado potato beetle, *Leptinotarsa decemlineata* (Coleoptera: Chrysomelidae). Sci. Rep. 8:1931. 10.1038/s41598-018-20154-1, PMID: 29386578 PMC5792627

[ref36] Shi X. -Q. Guo W. -C. Wan P. -J. Zhou L. -T. Ren X. -L. Ahmat T.. (2013). Validation of reference genes for expression analysis by quantitative real-time PCR in *Leptinotarsa decemlineata* (say). BMC. Res. Notes 6:93. 10.1186/1756-0500-6-93, PMID: 23497596 PMC3600673

[ref37] Shi Y. Jiang H. B. Gui S. H. Liu X. Q. Pei Y. X. Xu L.. (2017). Ecdysis triggering hormone signaling (ETH/ETHR-A) is required for the larva-larva ecdysis in *Bactrocera dorsalis* (Diptera: Tephritidae). Front. Physiol. 8:587. 10.3389/fphys.2017.00587, PMID: 28878684 PMC5572281

[ref38] Shi Y. Liu T. Y. Jiang H. B. Liu X. Q. Dou W. Park Y.. (2019). The ecdysis triggering hormone system, via ETH/ETHR-B, is essential for successful reproduction of a major pest insect, *Bactrocera dorsalis* (Hendel). Front. Physiol. 10:151. 10.3389/fphys.2019.00151, PMID: 30936833 PMC6431669

[ref39] Takahashi A. (2013). Pigmentation and behavior: potential association through pleiotropic genes in *Drosophila*. Genes Genet. Syst. 88, 165–174. 10.1266/ggs.88.165, PMID: 24025245

[ref40] Terhzaz S. O’Connell F. C. Pollock V. P. Kean L. Davies S. A. Veenstra J. A.. (1999). Isolation and characterization of a leucokinin-like peptide of *Drosophila melanogaster*. J. Exp. Biol. 202, 3667–3676. PMID: 10574744 10.1242/jeb.202.24.3667

[ref41] Truman J. W. (2005). Hormonal control of insect ecdysis: endocrine cascades for coordinating behavior with physiology. Vitam. Horm. 73, 1–30. 10.1016/S0083-6729(05)73001-6, PMID: 16399406

[ref42] Wittkopp P. J. Beldade P. (2009). Development and evolution of insect pigmentation: genetic mechanisms and the potential consequences of pleiotropy. Semin. Cell Dev. Biol. 20, 65–71. 10.1016/j.semcdb.2008.10.002, PMID: 18977308

[ref43] Wittkopp P. J. Stewart E. E. Arnold L. L. Neidert A. H. Haerum B. K. Thompson E. M.. (2009). Intraspecific polymorphism to interspecific divergence: genetics of pigmentation in *Drosophila*. Science 326, 540–544. 10.1126/science.1176980, PMID: 19900891

[ref44] Xu Q. -Y. Deng P. Li A. Zhang Q. Mu L. -L. Fu K. -Y.. (2019). Functional characterization of ultraspiracle in *Leptinotarsa decemlineata* using RNA interference assay. Insect Mol. Biol. 28, 676–688. 10.1111/imb.12580, PMID: 30834617

[ref45] Xu Q. -Y. Deng P. Zhang Q. Li A. Fu K. -Y. Guo W. -C.. (2020). Ecdysone receptor isoforms play distinct roles in larval-pupal-adult transition in *Leptinotarsa decemlineata*. Insect Sci. 27, 487–499. 10.1111/1744-7917.12662, PMID: 30688001 PMC7277042

[ref46] Xu Q. -Y. Meng Q. -W. Deng P. Guo W. -C. Li G. -Q. (2018). *Leptinotarsa* hormone receptor 4 (HR4) tunes ecdysteroidogenesis and mediates 20-hydroxyecdysone signaling during larval-pupal metamorphosis. Insect Biochem. Mol. Biol. 94, 50–60. 10.1016/j.ibmb.2017.09.012, PMID: 28951206

[ref47] Žitňan D. Kim Y. J. Žitňanová I. Roller L. Adams M. E. (2007). Complex steroid-peptide-receptor cascade controls insect ecdysis. Gen. Comp. Endocrinol. 153, 88–96. 10.1016/j.ygcen.2007.04.002, PMID: 17507015 PMC4955941

[ref48] Žitňan D. Ross L. S. Žitňan I. Hermesman J. L. Gill S. S. Adams M. E. (1999). Steroid induction of a peptide hormone gene leads to orchestration of a defined behavioral sequence. Neuron 23, 523–535. 10.1016/S0896-6273(00)80805-3, PMID: 10433264

