# Supplementary material for: Two Splice Isoforms of Leptinotarsa Ecdysis Triggering Hormone Receptor Have Distinct Roles in Larva-Pupa Transition
Source: Front Physiol. 2020 Dec 1;11:593962. doi: 10.3389/fphys.2020.593962 (PMC7736071; doi:10.3389/fphys.2020.593962)
Supplement: Supplementary file 1 [file Presentation_1.pdf]

Supplementary data

Two splice isoforms of *Leptinotarsa* ecdysis triggering hormone receptor have distinct roles in larva-pupa transition

Chen-Hui Shen[1], Qing-Yu Xu[1], Kai-Yun Fu[2], Wen-Chao Guo[3], Lin Jin[1]*, Guo-Qing Li [1]

1. Education Ministry Key Laboratory of Integrated Management of Crop Diseases and Pests, College of Plant Protection, Nanjing Agricultural University, Nanjing 210095, China
2. Institute of Plant Protection, Xinjiang Academy of Agricultural Sciences; Urumqi 830091, China
3. Institute of Microbiological Application, Xinjiang Academy of Agricultural Science; Urumqi 830091, China

Table S1. Primers used in RT-PCR, dsRNA synthesis and qPCR

Fragment name	Forward primer	Reverse primer
**RT-PCR**		
Ldethra	AACACAACCACATCCCACTATC	TGTCATGGCTACTTCTGAAGGT
Ldethrb	CATCCCACTATCTTTACACCCC	GCTTCTTCCTACACCTGATTCG
**dsRNA synthesis**		
ds*ethr*	TGAACACAACCACATCCC	GAGTTGACCTCGACAAGC
ds*ethra*	GGTCTGCCCTCTACTTCC	TGTAATACTTCTCAACGCCTA
ds*ethrb*	GGTTGGAGCCTTGAAATC	TCTTGAGAAAACTATCGGAA
ds*PTTH*	TTGCTTCTAGTAACAACCCA	GAGTCTCAGATAATGGTCGG
ds*SHD*	CTCTTCCTCGGTTATTCTTGCC	ATGCAAACCAGTTCAGGCC
ds*EcR*	GATCTATCCCCTCCCAGCAG	TCGTTCCGTTTGACAGCG
ds*USP*	AATCAATAAGTCCACCGC	CCAGACACTTCAAACCGA
ds*FTZ-F1*	TCTGCTGAGTTTGGGGTT	GTGCTTGTGGTAGAGGTGTT
ds*egfp*	AAGTTCAGCGTGTCCG	CACCTTGATGCCGTTC
**qPCR**		
q*Ldethr*	ACAGCAATGTCACCAACACGACA	TCAGCAACGCTCAAGTTCACCA
q*Ldethra*	TTTACGAGCCCAATGATAGGCAT	AGGAAGTAGAGGGCAGACCA
q*Ldethrb*	CCAGCAGGTTGGAGCCTTGA	TCCGGCTTCTTCCTACACCTGA
q*LdHR3*	GCATCATCCAGCAAATCATC	CTGATCGTCTTGCGACAACT
q*LdE75*	CCAACTCCAGATGCAGCTTA	AATGATTTCGCCAACATTGA
q*LdSHD*	GGCCTGAACTGGTTTGCAT	GGCAAGAATAACCGAGGAAGAG
q*LdEcR*	GAATGAGGGCAGAGTGTGTG	TCGTAGTGCTATTGGGCTTG
q*LdFTZ-F1*	GGCTAATCAGGCCTCCAG	CATGGTTTGCTGGCAACTAC
q*Ldbur*	CCTGTATGTGCTGCCAAGAA	GATTTGAGGAAATGGCCAGA
q*Ldpbur*	ACCTGTGAAACGTTGCCTTC	ACCATCTGGGTCGTAGCAGT
q*Ldth*	AAGACCGCCCTCCAGCCTTA	ACCTCGAAGGGCCTGGACAT
q*Ldddc*	CCAACAGGGCTCTGCTCCAG	TGCCGTACAGCCTTAACACGAA
q*Ldppox1*	CCGTTCGACAGGCAACCTAGA	CTCGCCTTTGGTGGTTCCGT
q*Ldppox2*	TTCGAGCGTCTCTGCCACAA	CGAGCTGGCCAAGTTCTGCT
q*Ldppox3*	ATGCGCAATGTGGACGACCT	TCTTGCGTGTCTGGGCGATG
q*LdRP18*	TAGAATCCTCAAAGCAGGTGGCGA	AGCTGGACCAAAGTGTTTCACTGC
q*LdRP4*	AAAGAAACGAGCATTGCCCTTCCG	TTGTCGCTGACACTGTAGGGTTGA
q*LdARF1*	CGGTGCTGGTAAAACGACAA	TGACCTCCCAAATCCCAAAC
q*LdARF4*	GTGCTCGTGAACCATGTGAA	AACCTCCAATCCCTCGTGAA

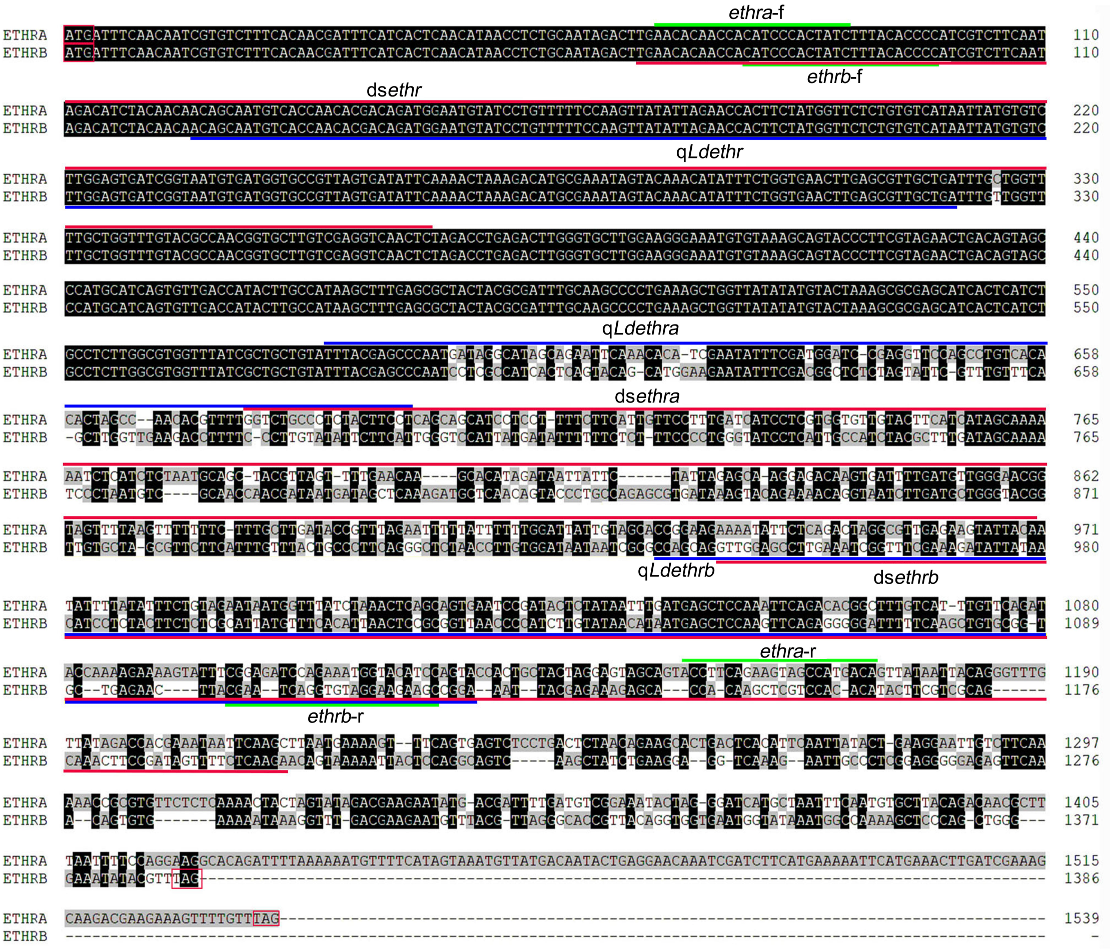

Figure S1. Alignment of nucleic acid sequences of *Ldethr* isoforms from *Leptinotarsa decemlineata*. Two *Ldethr* isoforms are aligned. Start and stop codons are shown by red boxes. The primers for RT-PCR are indicated with green lines; the sequences for measuring the expression levels of *Ldethr*, *Ldethra* and *Ldethrb* using qRT-PCR are marked with blue lines; and the sequences of ds*ethr*, ds*ethra* and ds*ethrb* used to respectively knock down *Ldethr*, *Ldethra* and *Ldethrb* are highlighted with red lines.

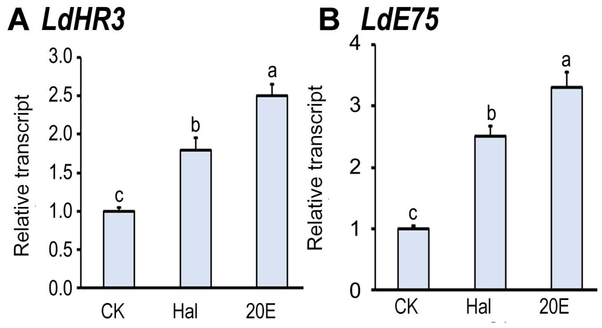

Figure S2. Ingestion of an ecdysteroid agonist halofenozide (Hal) and 20-hydroxyecdysone (20E) by fourth instar larvae affects the expression of early ecdysone response genes in *Leptinotarsa decemlineata*. For 1 day, the newly-ecdysed fourth-instar larvae have ingested potato foliage treated with water (control), 100 ng/mL of Hal, or 20E. The expression levels of *LdHR3* (A) and *LdE75* (B) were measured. Relative transcripts are the ratios of relative copy numbers in treated individuals to PBS-fed controls (CK), which are set as 1. The bars represent values (± SE). Different letters indicate significant difference at P value < 0.05 compared with ANOVA and the Tukey-Kramer test.

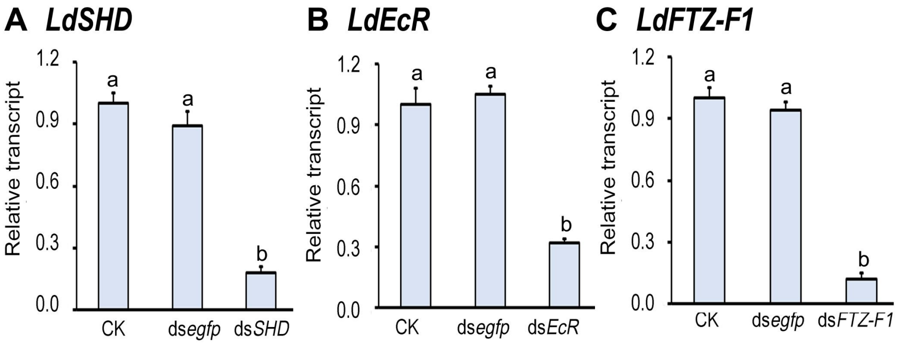

Figure S3. Ingestion of dsRNA by fourth instar larvae represses the expression of target gene in *Leptinotarsa decemlineata*. The newly-molted fourth-instar larvae have ingested ds*SHD*, ds*EcR* and ds*FTZ-F1* dipped leaves for 3 days. The larvae feeding PBS (CK) and ds*egfp* immersed foliage were used as control. The expression levels of *LdSHD* (A), *LdEcR* (B) and *LdFTZ-F1* (C) were determined. Relative transcripts are the ratios of relative copy numbers in treated individuals to PBS-fed controls (CK), which are set as 1. The bars represent values (± SE). Different letters indicate significant difference at P value < 0.05 compared with ANOVA and the Tukey-Kramer test.

A

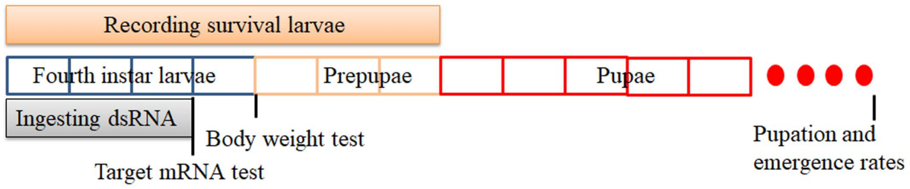

B

4th instar larvae	1-day-old	2-day-old	3-day-old	4-day-old
PBS	PBS	PBS	PBS	Normal foliage
dsegfp	dsegfp	dsegfp	dsegfp	Normal foliage
dsethra	dsethra	dsethra	dsethra	Normal foliage
dsethra+20E	dsethra	dsethra	dsethra+20E	Normal foliage

Figure S4. Summary of the experimental procedure timing in *Leptinotarsa decemlineata*. (A). The newly-ecdysed fourth instar larvae had ingested PBS (CK)-, ds*egfp*-, and ds*ethr*- (ds*ethra*- or ds*ethrb*-) dipped leaves for three days, and untreated foliage for an addition of one day. The expression levels of target mRNAs and the fresh larval weights were measured three and four days after the initiation of the bioassay. The survivorship was observed through the fourth-instar larvae and prepupae. The pupation and emergence rates were recorded during a 4-week trial period. (B). The feeding schedule in the rescuing bioassay. The larvae were firstly allowed to ingest potato foliage immersed with PBS (CK), ds*egfp* or ds*ethra* (ds*ethrb*) for two days, and then to consume leaves dipped with PBS, ds*egfp*, ds*ethra* (ds*ethrb*), ds*ethra* (ds*ethrb*)+20E, for an additional day. The larvae were then transferred to untreated foliage.
